# Association Between Social Network and Cognitive Function: A Cross-Sectional Assessment From the Cardiovascular and Metabolic Diseases Etiology Research Center Cohort (2013–2018)

**DOI:** 10.3389/fpsyt.2022.893290

**Published:** 2022-06-06

**Authors:** Jimin Kim, Ji Su Yang, Yoosik Youm, Dae Jung Kim, Hyeon Chang Kim, Sun Jae Jung

**Affiliations:** ^1^Department of Public Health, Graduate School, Yonsei University, Seoul, South Korea; ^2^Department of Sociology, Yonsei University, Seoul, South Korea; ^3^Department of Endocrinology and Metabolism, Ajou University School of Medicine, Suwon, South Korea; ^4^Department of Preventive Medicine, Yonsei University College of Medicine, Seoul, South Korea

**Keywords:** social network size, social network intimacy, frequency of meeting, cognitive function, public health

## Abstract

**Background:**

This study aimed to investigate how social networks are associated with cognitive function in the middle-aged and elderly Korean population.

**Methods:**

A total of 7,704 individuals over the age of 50 were included from the baseline recruitment of the Cardiovascular and Metabolic Diseases Etiology Research Center cohort from the years 2013 to 2018. Egocentric social network characteristics including network size, intimacy, and frequency of face-to-face meetings were measured as exposures, and the Korean version of Mini-Mental State Examination (K-MMSE) score was measured to reflect general cognitive function as an outcome. We also stratified the analysis by income level into tertiles, with income caps of 42,000 thousand won and 72,000 thousand won. A general linear regression model was used, adjusting for age, gender, socioeconomic factors, lifestyle factors, depressive symptoms, and study settings.

**Results:**

Social network properties were positively associated with cognitive function in both men and women. However, the specific estimates varied according to gender and income level. In men, frequency was most significantly associated with cognitive function (standardized β = 0.093, *p*-value <0 .0001). In women, the strength of the association with cognitive function was found in size (standardized β = 0.055, *p*-value = 0.001). The effect modification of income level could be seen in the association between frequency and cognitive function. The strongest association between frequency and cognitive function was found in the middle income group in men (standardized β = 0.114, *p*-value = 0.0063), and the low income group in women (standardized β = 0.076, *p*-value = 0.0039).

**Conclusion:**

There were positive associations between social network properties (i.e., size, intimacy, and frequency of face-to-face meetings) and cognitive function. The degree of association varied according to social network properties, gender, and income level. Overall, among social network properties, social network size was an important factor in the cognition of women, whereas frequency was important in the cognition of men.

## Introduction

Dementia is a chronic disease characterized by the impairment of cognitive functions such as thinking, memory, and reasoning. It is one of the greatest global challenges of the twenty-first century impacting health and social welfare (1). Moreover, the rise in life expectancy seems to have contributed to the increased prevalence of dementia (2). It has been estimated that there were more than 50 million people with dementia worldwide in 2019, and this number is expected to increase to 152 million by 2050 (3). In fact, the prevalence of dementia among Koreans aged 65 years and older was 9.2%, exceeding the estimated prevalence in other Asian countries (4.19–7.63%) (4). Further, the prevalence and incidence of dementia in Korea increased from 2003 to 2015 (5). As dementia requires continuous care and medical expenses, which are higher in cost than other diseases, its economic burden is difficult to ignore (6). The global cost of dementia in 2015 ($818 billion) has increased by 35.4% from that in 2010 ($604 billion) and is anticipated to reach $2 trillion by 2030 (7). Furthermore, in Korea, as of 2015, the elderly with dementia spent 2.4 times more in lifetime medical expenditures ($65,427) than elderly without dementia ($26,439) (6).

Cognitive function has been known to be related to socioeconomic status, activities of daily living, depressive symptoms, and social networks (8). Over the decades, several studies have investigated the relationship between social networks and cognitive function. According to a systematic review of longitudinal studies, a lack of social relationships increased cognitive decline (9). This study classified social relationships by structural (e.g., social network size, social activity) and functional (e.g., social support) aspects, and conducted the meta-analysis independently. Both aspects of social relationships had protective effects on cognitive decline (structural, OR = 1.08; functional, OR = 1.15) (9). However, the effect of social relationships on cognition may have been overestimated due to publication bias (9). Another meta-analysis of 39 articles showed that social activity had positive associations with global cognition, working memory, and processing speed but not with episodic memory or attention (10). Social network size and frequency of contact were associated with better global cognition and episodic memory but not with processing speed or attention (10). However, the results were not consistent between the studies because there were differences in the characteristics of the studies such as target population and study design (10).

Several longitudinal studies have reported that a better social network (e.g., network size and social activities) at baseline was associated with higher initial cognitive performances, but the association with subsequent cognition remains controversial (11, 12). The association with cognition was different depending on the network types (13). Individuals with multiple sources of networks (e.g., friends and relatives) had better cognitive outcomes, while network ties mainly with relatives had poorer cognitive health. A study in community-dwelling Spanish older adults reported that having a larger social network size (i.e., the number of family or friends with whom they had recent contact) decreased the risk of cognitive decline in the elderly (14). Another study in Taiwan suggested that participation in social activities reduced cognitive impairment as an important predictive factor (8). Other factors, such as marital status, living arrangement, emotional support, frequency of contact, and satisfaction, seemed to be associated with cognitive ability in old age, as seen in several studies (15–19). This pattern was not considerably different from that observed in studies conducted in South Korea. According to one study based on the Korean Longitudinal Study of Aging (KLoSA), older adults who were living with their spouses and had frequent contact with their children had a lower risk of cognitive decline (20). Moreover, other studies using KLoSA data showed that participating in diverse social activities had a positive effect on preventing cognitive impairment (21, 22).

Several previous studies have explored how social network size, social activities, and social engagement affect cognitive function in the elderly in many countries. However, most studies in Korea have focused more on social activities rather than social network properties, such as size or intimacy. Further, most studies were analyzed without classifying men and women. We expect that the association between social network properties and cognitive function would differ according to gender; however, evidence of this is insufficient. Moreover, research on the effect modification of socioeconomic status on the association is scarce. Therefore, we aimed to investigate the association between social network properties and cognitive function according to gender and income level in middle-aged and elderly South Korean population.

## Materials and Methods

### Data Collection and Participants

This study was designed as a cross-sectional study and used data from the Cardiovascular and Metabolic Diseases Etiology Research Center (CMERC) cohort. The CMERC cohort was designed to investigate the risk factors for cardiovascular disease (CVD) and produce evidence regarding the preventive treatment of CVD (18). Community-dwelling adults who did not have cardiovascular diseases were recruited by two research clinics (Yonsei University College of Medicine in Seoul and Ajou University School of Medicine Suwon), and hospital-based population participants were recruited at Severance Hospital, Seoul (23). The YUCM Clinic recruited eligible participants from Seoul, and capital areas including Incheon, Goyang, and Gimpo. The AUSM Clinic registered individuals living in Suwon, Yongin, and Hwaseong, which are the southern part of the capital area (23). The participants were recruited through advertisements in newspapers or posters in public places (23). Baseline measurements included socio-demographic factors, medical history, health-related behaviors, psychological health, social network, and support, which were collected through face-to-face interviews (23). Physical examinations including blood analysis and urinalysis were also conducted (23). A total of 8,697 community-based participants aged 30–64 years and 3,267 hospital-based participants aged 30–80 years were included in the baseline assessment between 2013 and 2018 (24). We excluded individuals under the age of 50 (*N* = 3,659) and those who had missing data on social network properties (*N* = 151) and Mini-Mental State Examination (*N* = 450). A total of 7,704 individuals (i.e., 3,027 men and 4,677 women) were included in the final analysis.

### Exposure Variables: Social Network Properties

Information about egocentric social networks including network size, density, content, composition, intimacy, the volume of contact, and bridging potential (network member only connected to the respondent), was collected through face-to-face interviews by trained interviewers (23, 25). These factors were assessed using the same questionnaire developed by the US National Social Life, Health, and Aging Project (NSHAP) which collected the list of multiple network members associated with the respondents and the relationships with them (23, 26). Social network properties were measured using the name generator module, which had been developed for the General Social Survey in the United States (25). The Korean version was previously carried out in a nationwide social survey and validated to consistently measure key networks in health research (26). The same questionnaires were used in the Korean Social Life, Health, and Aging Project (KSHAP) (23). We used social network size, intimacy and volume of contact to represent the social network status of participants. For network size, we asked participants to list up to six people, including their spouse, with whom they had discussed any important matters during the last 12 months. Since network size members are those who had discussed “important matters” with the participants, this measure captures the type of social bonds that are most likely to provide social support (25). The social network size is the core confidantes and is regarded important when social influence and support are considered (25). Next, they were asked “How close do you feel your relationship is with [name]?” and were asked to respond using a 4-point Likert scale. Possible responses included “not very close” (1), “somewhat close” (2), “very close” (3), and “extremely close” (4). Subsequently, we averaged the total score of all responses for network members and named this value “intimacy.” Finally, participants answered how often they would meet each member of their network, by using an 8-point Likert scale ranging from “less than once a year” (1), “once a year” (2), “more than once a year” (3), “once a month” (4), “once every 2 weeks” (5), “once a week” (6), “more than once a week” (7), and “everyday” (8). Likewise, we averaged the total score of all responses for network members and named this value “frequency.” It did not include the contact by phone or text and only the face-to-face meeting was measured. The social network card used in the CMERC study (24) is described in Supplementary Table 1.

### Outcome Variable: Cognitive Function

In this study, the Korean version of the Mini-Mental State Examination (K-MMSE) was conducted on participants over 50 years of age to assess global cognitive function. The MMSE is a widely used examination for screening dementia in both population studies and clinical practice (27). K-MMSE comprises 30 questions on orientation, verbal memory, concentration and calculation, language, praxis, and visuospatial construction. The estimated Cronbach’s alpha of the K-MMSE was 0.84, and that for the normal population was 0.74 (28). Further, the sensitivity was 0.91, and the specificity was 0.76 (28). Although the cut-off score of 24 is conventionally used for screening cognitive deficit, individuals with a score of 24 and under were few among our participants. Considering the statistical power, we used the total score as a continuous variable in the analysis.

### Covariates

Covariates were age, gender, socioeconomic factors, lifestyle factors, depressive symptoms, and study settings. Socioeconomic factors included household income, years of education, marital status, living arrangements, and occupation. We classified the household income level into tertiles: a yearly income of 42,000 thousand won and under as a low-income group, over 42,000 thousand won and less than 72,000 thousand won as a middle-income group, and over 72,000 thousand won as a high-income group. Years of education were categorized into four groups according to the final WSA education level: 6 years or lower, 7 years to 9 years, 10 years to 12 years, and more than 12 years. Marital status was classified as married, divorced including separated, widowed, or never married. Living arrangement was dichotomized as living alone or not, and occupation as employed or not. Students and housewives were included in the employed group. Lifestyle factors included cigarette use, alcohol consumption, and physical activity. Cigarette use and alcohol consumption were categorized as current, past, or never used. For physical activity, a Korean version of the International Physical Activity Questionnaire-Short Form (29) was used, and participants were asked about the number of days and duration of three levels of exercise: vigorous intensity activities, moderate intensity activities, and walking during the last 7 days. Based on this information, we quantified the total score of physical activity in MET minutes. We weighted 3.3 METs for walking, 4.0 METs for moderate intensity activities, and 8.0 METs for vigorous intensity activities (30). Depressive symptoms during the past 2 weeks were measured using the Beck Depression Inventory-II, which has been verified in the Korean general population (31). Since the data was collected from three different settings in two study centers (23, 32), study setting was included as a covariate.

### Statistical Analysis

We analyzed baseline characteristics classified by K-MMSE score and gender to check for differences between the groups using the chi-square test and independent *t*-test. The association between social networks and cognitive function stratified by gender was analyzed using a general linear regression model. The model for estimating the effect of social network properties is


$$\text{Y}=\beta_{0}+\beta_{1}\,x_{1}+\beta_{2}\,x_{2}+\beta_{3}\,x_{3}+\cdots +\beta_{14}\,x_{14}+\varepsilon$$

where Y denotes the K-MMSE score, β0 is the intercept term, and βn is the regression coefficient of the independent variable xn (x1 = social network size; x2 = intimacy; x3 = frequency; x4 = age; x5 = household income; x6 = education; x7 = marital status; x8 = living arrangement; x9 = occupation; x10 = physical activity (MET-minutes); x_11_ = Beck Depression Inventory-II score; x_12_ = cigarette use; x_13_ = alcohol consumption; x_14_ = study setting). Further, we standardized the estimates to compare the strength of the associations among social network properties. The confounders were selected by reviewing prior studies (8, 33, 34). Model 1 was adjusted for age and model 2 was additionally adjusted for socioeconomic factors. The final model was additionally adjusted for lifestyle factors, depressive symptoms and study settings. As the economic condition can act as an effect modifier, we also stratified the income level into tertiles and conducted the analysis using the same models. As a sensitivity analysis, logistic analysis was performed with K-MMSE score of over 24 as the outcome. All statistical analyses were performed using SAS version 9.4 (SAS Institute Inc., Cary, NC, United States).

## Results

### Characteristics of Study Population

The baseline characteristics of the study population are described in Table 1. We classified individuals into “low cognitive function (case)” for those who had K-MMSE 24 or under; the remaining participants were categorized as “reference” in each gender. “Frequency” in both genders are between 1 and 2.5 which seems low, but this result could be due to the exclusion of contact by phone or text messages. In men, there was no significant age difference between the case and reference groups. In women, the cases were slightly older (59.0 y ± 6.17) than the references (58.33 y ± 5.88). In both men and women, there were higher proportions of low-income and low-education levels (6 years and under) in the case than in the reference group. There were no significant differences in marital status among men. However, in women, the reference showed a higher proportion of married women than the case, while the proportion of bereaved individuals was higher in the case. The proportion of physical activity and depression was higher in the case than in the reference in both men and women. When comparing the characteristics between men and women, there were several noticeable differences. The proportion of the case group was greater in women (22.2%) than in men (14.7%). Also, women were more likely to have lower educational levels, be widowed, live alone, and have a job than men.

**TABLE 1:** General characteristic of the cardiovascular and metabolic diseases etiology research cohort (CMERC) participants (*N* = 7,704).

Characteristic		Men (*N* = 3,027)		*p*-value	Women (*N* = 4,677)		*p*-value
				
		K-MMSE ≤ 24 (*N* = 445)	K-MMSE > 24 (*N* = 2,582)		K-MMSE ≤ 24 (*N* = 1,036)	K-MMSE > 24 (*N* = 3,641)	
Age, mean ± *SD*		59.65 ± 6.66	59.97 ± 6.63	0.3455	59.0 ± 6.17	58.33 ± 5.88	0.0013
Income, (10,000W = /year), median (Q1–Q3)		6,000 (3,600–7,800)	6,000 (4,200–9,600)	<0.0001	4,800 (3,000–7,200)	6,000 (3,600–8,400)	<0.0001
	Low	145 (35.89%)	605 (26.52%)	<0.0001	453 (49.13%)	1,143 (34.28%)	<0.0001
	Middle	151 (37.38%)	811 (35.55%)		280 (30.37%)	1,137 (34.10%)	
	High	108 (26.73%)	865 (37.92%)		189 (20.50%)	1,054 (31.61%)	
	N/A	41	301		114	307	
Education years, *N* (%)	≤6 years	59(13.29%)	152 (5.89%)	<0.0001	250 (24.13%)	409 (11.24%)	<0.0001
	6–9 years	65 (14.64%)	211 (8.17%)		262 (25.29%)	516 (14.18%)	
	9–12 years	196 (44.14%)	920 (35.63%)		416 (40.15%)	1,721 (47.28%)	
	>12 years	124 (27.93%)	1,299 (50.31%)		108 (10.42%)	994 (27.31%)	
	N/A	1	0		0	1	
Marital status, *N* (%)	Not married	1 (0.22%)	14 (0.54%)	0.1136	6 (0.58%)	60 (1.65%)	0.0043
	Married	437 (98.20%)	2,476 (95.89%)		842 (81.27%)	3,011 (82.70%)	
	Widowed	1 (0.22%)	27 (1.05%)		119 (11.49%)	319 (8.76%)	
	Divorced	6 (1.35%)	65 (2.52%)		69 (6.66%)	251 (6.89%)	
Living arrangement, *N* (%)	Living alone	6 (1.35%)	46 (1.78%)	0.6512	72 (6.96%)	224 (6.15%)	0.3868
	Have cohabitants	439 (98.65%)	2,536 (98.22%)		963 (93.04%)	3,417 (93.85%)	
	N/A	0	0		1	0	
Occupation, *N* (%)	Employed	347 (77.98%)	1,988 (77.02%)	0.703	987 (95.27%)	3,473 (95.39%)	0.9423
	Unemployed	98 (22.02%)	593 (22.98%)		49 (4.73%)	168 (4.61%)	
	N/A	0	1		0	0	
Cigarette use, *N* (%)	Never	90 (20.22%)	548 (21.24%)	0.0156	1,003 (96.81%)	3,502 (96.24%)	0.6621
	Past	235 (52.81%)	1,493 (57.87%)		19 (1.83%)	82 (2.25%)	
	Current	120 (26.97%)	539 (20.89%)		14 (1.35%)	55 (1.51%)	
	N/A	0	2		0	2	
Alcohol consumption, *N* (%)	Never	59 (13.26%)	321 (12.45%)	0.5656	483 (46.67%)	1,584 (43.53%)	0.0049
	Past	53 (11.91%)	353 (13.69%)		19 (1.84%)	135 (3.71%)	
	Current	333 (74.83%)	1,905 (73.87%)		533 (51.50%)	1,920 (52.76%)	
	N/A	0	3		1	2	
Physical activity, (min-METS/week), median (Q1–Q3)		1,173 (396–2,613)	1,386 (594–3,066)	<0.0001	693 (264–1638)	1,173 (490–2,385)	<0.0001
Depression (BDI), (range: 0–63), median (Q1–Q3)		9 (5–15)	7 (3–13)	<0.0001	11 (6–18)	10 (6–15)	<0.0001
Social network size, (range: 0–6), median (Q1–Q3)		2 (1–3)	2 (1–4)	<0.0001	3 (2–4)	3 (2–5)	<0.0001
Intimacy, (range: 1–4), median (Q1–Q3)		3.0 (2.8–4.0)	3.33 (3.0–4.0)	<0.0001	3.0 (2.67–3.67)	3.17 (2.8–3.75)	0.0002
Frequency, (range: 1–8), median (Q1–Q3)		1.0 (1.0–2.5)	2.0 (1.0–3.0)	<0.0001	2.0 (1.0–3.0)	2.5 (1.5–3.33)	<0.0001
K-MMSE, median (Q1–Q3)		23 (22–24)	28 (27–29)	<0.0001	23 (21–24)	27 (26–29)	<0.0001

*BDI, Beck Depression Inventory-II; K-MMSE, Korean version of Mini-Mental State Examination.*

### Association Between Social Network Properties and Cognitive Function Stratified by Gender

The gender-stratified associations of social network properties and cognitive function are shown in Table 2. In both men and women, larger social network status was associated with higher cognitive function. In men, frequency showed the greatest association with cognitive function among three social network properties. In the final model, as one standard deviation of frequency increased, the K-MMSE score increased by 0.093 (standardized β = 0.093, standard error [SE] = 0.050, *p*-value < 0.0001); one standard deviation of size increased the K-MMSE score by 0.058 (standardized β = 0.058, SE = 0.037, *p*-value = 0.0181), and one standard deviation of intimacy increased it by 0.047 (standardized β = 0.047, SE = 0.074, *p*-value = 0.0122). In women, among the three properties, size was mostly associated with cognitive function. In the final model, one standard deviation of size increased the K-MMSE score by 0.055 (standardized ß = 0.055, SE = 0.028, *p*-value = 0.0010); one standard deviation of frequency increased it by 0.051 (standardized β = 0.051, SE = 0.034, *p*-value = 0.0013), and one standard deviation of intimacy increased it by 0.032 (standardized ß = 0.032, SE = 0.063, p-value = 0.0305). Bonferroni correction was applied for multiple comparisons of the social network properties (significance *P*-values < 0.0167).

**TABLE 2:** Associations between social network properties and K-MMSE score in cardiovascular and metabolic diseases etiology research cohort (CMERC) participants.

Men	Model 1 (*N* = 3,027)				Model 2 (*N* = 2,685)				Model 3 (*N* = 2,678)			
			
	β	*Std*β	SE	*p*-value	β	*Std*β	SE	*p*-value	β	*Std*β	SE	*p*-value
Size (0–6)	0.114	0.076	0.034	0.0009*	0.096	0.064	0.035	0.0068*	0.087	0.058	0.037	0.0181
Intimacy (1–4)	0.367	0.095	0.069	<0.0001*	0.221	0.056	0.073	0.0025*	0.186	0.047	0.074	0.0122*
Frequency (1–8)	0.234	0.113	0.047	<0.0001*	0.188	0.090	0.050	0.0002*	0.194	0.093	0.050	<0.0001*

**Women**	**Model 1 (*N* = 4,677)**				**Model 2 (*N* = 4,255)**				**Model 3 (*N* = 4,252)**			
			
	β	***Std*β**	**SE**	***p*-value**	β	***Std β***	**SE**	***p*-value**	β	***Std β***	**SE**	***p*-value**

Size (0–6)	0.228	0.132	0.027	<0.0001*	0.197	0.117	0.027	<0.0001*	0.093	0.055	0.028	0.001*
Intimacy (1–4)	0.313	0.073	0.061	<0.0001*	0.108	0.026	0.063	0.0827	0.136	0.032	0.063	0.0305
Frequency (1–8)	0.158	0.072	0.033	<0.0001*	0.121	0.056	0.035	0.0005*	0.111	0.051	0.034	0.0013*

*Model 1 : adjusted for age.*

*Model 2 : adjusted for age, household income, education level, marital status, living arrangement and occupation.*

*Model 3: adjusted for age, household income, education level, marital status, living arrangement, occupation, physical activity (MET-minutes), BDI score, cigarette use, alcohol consumption, and study setting.*

*K-MMSE, Korean version of Mini-Mental State Examination; Std β, standardized β; SE, standard error.*

*Significant in Bonferroni correction.

The odds ratios (ORs) for K-MMSE score of over 24 are shown in Supplementary Table 2. In men, as one unit of frequency increases, the OR for K-MMSE score of over 24 was 1.213 (95% CI = 1.062–1.384). In women, as one unit of network size increases, the OR for K-MMSE score of over 24 was 1.088 (95% CI = 1.027–1.154). These results correlates with our main results (Table 2) that the frequency is more significantly associated with K-MMSE in men, and size is more significantly associated with K-MMSE in women.

### Association Between Social Network Properties and Cognitive Function Stratified by Income Level

The income-stratified associations between social network properties and cognitive function are shown in Table 3. First, we tested whether there were interaction effects between social networks and income. The interaction effect was found only between frequency and income (men, *p*-interaction = 0.0461; women, *p*-interaction = 0.0183). In men, middle income group showed the strongest association between frequency and cognitive function (low income, standardized β = 0.107, *p*-value = 0.0211; middle income, standardized β = 0.114, *p*-value = 0.0063; high income, standardized β = 0.070, *p*-value = 0.0726). In women, frequency and cognitive function had the greatest association in low income group (low income, standardized β = 0.076, *p*-value = 0.0039; middle income, standardized β = 0.072, *p*-value = 0.0116; high income, standardized β = −0.017, *p*-value = 0.5699). Bonferroni correction was applied for multiple comparisons of the social network properties and income level (significance *P*-values < 0.0056).

**TABLE 3:** Associations between social network properties and K-MMSE score stratified by income level in cardiovascular and metabolic diseases etiology research cohort (CMERC) participants.

		Men					Women				
			
		*N*	β	*Std*β	SE	*p*-value	*N*	β	*Std*β	SE	*p*-value
		***p*-interaction = 0.1549**					***p*-interaction = 0.1240**				
Size (0–6)	Low income	746	0.124	0.075	0.079	0.1151	1,594	0.064	0.034	0.051	0.2037
	Middle income	959	0.030	0.020	0.068	0.6554	1,416	0.099	0.062	0.048	0.0388
	High income	973	0.113	0.089	0.051	0.0264	1,242	0.129	0.085	0.048	0.0069
		***p*-interaction = 0.1780**					***p*-interaction = 0.3064**				
Intimacy (1–4)	Low income	746	0.175	0.043	0.150	0.2434	1,594	0.266	0.060	0.110	0.0153
	Middle income	959	0.140	0.035	0.128	0.2767	1,416	0.065	0.016	0.106	0.5405
	High income	973	0.253	0.070	0.113	0.0256	1,242	0.016	0.004	0.110	0.8809
		***p*-interaction = 0.0461**					***p*-interaction = 0.0183**				
Frequency (1–8)	Low income	746	0.234	0.107	0.101	0.0211	1,594	0.149	0.076	0.052	0.0039*
	Middle income	959	0.248	0.114	0.091	0.0063	1,416	0.169	0.072	0.067	0.0116
	High income	973	0.131	0.070	0.073	0.0726	1,242	-0.038	-0.017	0.068	0.5699

*The model is adjusted for age, household income, education level, marital status, living arrangement, occupation, physical activity (MET-minutes), BDI score, cigarette use, alcohol consumption and study setting.*

*K-MMSE, Korean version of Mini-Mental State Examination; Std β, standardized β: SE, standard error.*

*Significant in Bonferroni correction.

## Discussion

These analyses of social network and cognitive function in the Korean elderly showed that the associations between social network size, intimacy, frequency and cognitive function vary according to gender and income level. In men, frequency was the most important factor associated with cognitive function, whereas in women, network size had the greatest association with cognitive function. These associations persisted after adjusting for potential confounders. Furthermore, there was an interaction effect of frequency and income on cognitive function. However, when stratified by income level, under Bonferroni correction, none of the income groups showed significant association between (8) frequency and cognitive function except low income group in women.

Although our results showed slight differences in specific estimates, in general, expanded social network size and higher frequency were positively associated with cognitive function. Few studies have been conducted in Korea on topics similar to ours. One study investigated which type of social activity affected cognitive decline using the KLoSA (21). Among 1,568 participants aged 65 years of age or older who participated in senior citizen clubs, who had frequent contact by phone or letters with offspring reduced cognitive decline. However, face-to-face contact with offspring showed a positive association with cognitive decline, which contradicts our results. This difference can be explained by the different study designs and demographic characteristics of participants. This study was a longitudinal study, and the association mentioned above was significant in adults aged 70 years or older. Face-to-face contact might not have a positive effect on cognitive function in the elderly and the specific individuals one meets may be a major factor influencing cognitive abilities.

The term “social network” refers to the total set of connections between all members of a particular individual, and networks can be classified into different dimensions (35). There are additional studies that have measured different social network variables. A study based on the Kungsholmen Project—a longitudinal population-based study on aging and dementia in 1987 in Sweden—included 1,203 participants who were 75 years or older without a diagnosis of dementia (16). They reported that frequent but unsatisfactory contact with children increased the incidence of dementia. They had fewer and older participants than our study. Unlike our study, they also measured the satisfaction of contact, which, therefore, could allow for investigation of the effect of both the quantitative network and qualitative network. Furthermore, some studies used an integrated index, such as the Lubben Social Network Scale, to assess the size and perceived support confidant network and claimed that a larger social network might benefit cognitive function (18, 36). Although there are several studies about the relationship between social network and cognition, more studies measuring diverse dimensions of social networks are necessary to examine the influence of different social network resources.

In terms of outcomes, various indicators were used to assess cognitive function. Some studies cited questions from the Short Portable Mental Status Questionnaire (8, 14, 37); other studies assessed cognitive function by the Moray House Test, Telephone Interview for Cognitive Status-modified, or used different tests for different sectors of cognitive ability (17, 18, 38). A study examining the association between social engagement and cognitive decline in African American adults, measured cognitive function by assessing several cognitive domains; the associations for each domain have been reported (39). Therefore, further study in Korea is needed to investigate the effect on specific sectors of cognitive function that are typically impaired in dementia.

Researchers have suggested possible mechanisms by which social networks may affect cognitive function. One possible pathway is related to stress. Stress and depression are known to affect brain function (18). Social support enhances a clear understanding of stress situations, helps to develop reasonable behaviors to deal with problems, and strengthens positive self-feelings (40). Through these processes, social support weakens the impact of stress which could affect cognition (40). Low social support is also known to be related to neuroendocrine and physiological factors of elevated stress reactivity (41). Social support may mitigate genetic and environmental vulnerabilities and strengthen resilience to stress via the hypothalamic-pituitary-adrenocortical system, noradrenergic system, and central oxytocin pathways (41). Thus, it affects neuroendocrine measures of stress, which may be linked to cognition (42). Another possible pathway is by improving healthy behaviors. By facilitating access to health care and healthy behaviors, social networks can indirectly reduce or prevent brain pathologies and other conditions related to cognition (18). Individuals with larger social networks have greater access to various resources or health-related information, which may lead to positive, healthy behaviors and prevent cognitive decline (43).

Social network size is also known to be related to social activity (33). According to a study examining the effect of social networks and activity on cognitive function, social activity participation showed a stronger association with cognition than social network resources which included social network size and frequency (44). However, the positive association of social activity attenuated as social network resources increased (44). In other words, for people who did not have close social ties, engaging in diverse social activities helped to strengthen positive correlation with cognitive recall. Thus, this finding indicates that social activities are beneficial to those with limited social network resources. A randomized-control trial in Finland reported the effect of social group intervention on cognitive function in elderly people (45). The participants participated in one of three types of group activities–writing, exercise, and art- once a week for 3 months (45). As the participants chose which social activity group to attend, they could form friendships with people of similar interests. They shared their feelings and experiences and received social support from one another. The intervention group showed more improved cognition than the control group (45). This result indicates that cognition is interrelated with social networks and activities. When the effect of productive activities (e.g., quilt, photo) and receptive activities (e.g., movies, games) on cognition was compared, episodic memory was more improved in the former group than in the latter group (46). Productive engagement can strengthen oneself and encourage continuous learning and intellectual stimulation (46). These results provide evidence that learning new things and concentrating one’s mind can be an important key to preventing cognitive decline.

Possible explanations for our finding that the degree of associations among social network properties differ based on gender and income may be found in previous studies. Evidence shows gender differences in social characteristics—men rely on their partners more exclusively for social support; women have greater social networks of friends and relatives from whom they can receive support (47). This supports our finding on the importance of frequency in men, and size in women. Another gender-stratified study in Korea reported that social network size and social activity had significant associations with cognition only in the female group (48). Although the participants were recruited in one rural area, the result that emphasizes the importance of social network size in women shows the same direction as our findings. One study that examined the effect of socioeconomic status on social networks reported that socioeconomic status—particularly education and occupation—affects network structure differentially among men and women (49). For example, men have more opportunities to form social connections in the workplace than women because women usually have lower-wage jobs in poor, isolated working conditions. The difference in socioeconomic level (e.g., income, occupation and education) between men and women may influence the composition of social networks and might lead to different effects of social networks on mental health, including cognitive function. However, it is difficult to clarify the mechanism of effect modification of income in our results because socioeconomic status is complexly correlated with each other, and gender might also play a significant role in this relationship. Therefore, further studies are needed to investigate the effect of socioeconomic status on the association between social networks and cognitive function.

Several limitations should be considered when interpreting our results. First, as the study design is cross-sectional, the result cannot be interpreted as a causal relationship, and the possibility of reverse causality should not be overlooked. Social network properties and K-MMSE score were measured in the same survey at the baseline of the cohort. Thus, there may be a possibility of changes in social networks as a consequence of cognitive decline. A longitudinal study is required to examine the effects of social network properties on cognitive function. Second, it is hard to say that our results are generalizable to the entire population since the participants were recruited from relatively urban areas. Also, the fact that social network properties can be affected by urbanicity might have influenced our results (50). A nationwide study of social networks could help investigate the effect of social networks and its association with cognitive function. Third, the MMSE only reflects cognitive status, not the diagnosis or incidence of dementia. Nor the past diagnosis of dementia could be known in our data source. Moreover, we could not determine the association with specific cognitive domains such as orientation, memory, concentration, and visuospatial construction. Fourth, social network properties were measured by individuals’ self-reported data, which can involve a risk of recall bias, and only egocentric network was measured. Also, a possibility of unmeasured close relationships exists. Since the network size was measured to be up to six people, social relationships with people beyond the network members were not included. However, since only 1.39% of our participants answered they had more than 6 network members, the potential for unmeasured close relationships might be small. Frequency was measured for only face-to-face meetings, so it did not reflect the case of phone calls or text messages. Finally, there might be an influence of unmeasured confounders due to the lack of data sources.

Despite these limitations, our study is meaningful and has several strengths. First, we considered a sufficient number of middle-aged and elderly participants. Unlike most studies on cognitive function and dementia, whose participants were 65 years or older, we included individuals aged 50 years and older. Second, we compared the associations by gender. Previous studies have suggested gender differences in the perception and relation of social networks and the influence of social ties on cognition. They reported that engagement with friends and social participation showed a protective effect on cognitive decline and mental health in women but not in men (14, 51). In another study, women had more close persons and reported greater satisfaction with their relationships than men, and men answered they receive higher levels of support from the closest person in their relationship (52). Our results support those previous studies that the relationship between social network and cognitive function have different characteristics according to gender, and offer a new perspective on this association. Third, we investigated the effect modification of income level on the association between social networks and cognitive function. As income is one of the most important factors related to cognitive function (53, 54), our results can serve as a basis for future research.

## Conclusion

Our study showed the association between three social network properties and cognitive function among middle-aged and elderly Koreans, which differed based on gender and income level. This study indicated the importance of social network size in women and the frequency of contact among men. It would be beneficial to encourage social relationships in older populations by supporting a variety of activities in community centers or establishing a space where the elderly can interact with each other. The individuals will be able to choose the group they are interested in, and maintain a stronger relationship with people with the same interests. Also, as our findings reported that frequency is important in men and network size is important in women, sufficient participants should be recruited and social meetings should be held frequently. Further studies are needed to investigate the effect of other social properties on cognitive function, and the causal relationship between them.

## Data Availability Statement

The original contributions presented in this study are included in the article/Supplementary Material, further inquiries can be directed to the corresponding author/s.

## Ethics Statement

The studies involving human participants were reviewed and approved by the Institutional Review Board of Yonsei University Health System, Seoul, Korea (4-2013-0661). The patients/participants provided their written informed consent to participate in this study.

## Author Contributions

JK: conceptualization, methodology, formal analysis, data curation, writing—original draft, writing—review, and editing. JSY: data curation, writing—review, and editing. YY, DJK, and HCK: writing—review and editing. SJJ: supervision, conceptualization, data curation, writing—original draft, writing—review, and editing. All authors contributed to the article and approved the submitted version.

## Conflict of Interest

The authors declare that the research was conducted in the absence of any commercial or financial relationships that could be construed as a potential conflict of interest.

## Publisher’s Note

All claims expressed in this article are solely those of the authors and do not necessarily represent those of their affiliated organizations, or those of the publisher, the editors and the reviewers. Any product that may be evaluated in this article, or claim that may be made by its manufacturer, is not guaranteed or endorsed by the publisher.
