# Supplementary material for: Association Between Social Network and Cognitive Function: A Cross-Sectional Assessment From the Cardiovascular and Metabolic Diseases Etiology Research Center Cohort (2013–2018)
Source: Front Psychiatry. 2022 Jun 6;13:893290. doi: 10.3389/fpsyt.2022.893290 (PMC9207251; doi:10.3389/fpsyt.2022.893290)
Supplement: Supplementary file 1 [file Table_1.docx]

Supplementary Table 1. Social network card used in Cardiovascular and Metabolic Diseases Etiology Research Cohort (CMERC) study

|  | | **E1. E2-1.**  **E2-2.**  **Name** | **E2-3.**  **Relation** | **E3.**  **Age** | **E4.**  **Sex** | **E5.**  **Education level** | **E6.**  **Are you living together with this person?** | **E6-1.**  **(If not)**  **Where does he/she live?** | **E7.**  **How long have you known each other?** | **E8.**  **How often do you talk to him/her?** | **E9.**  **How often do you meet him/her?** | **E10.**  **How close are you to him/her?** | **E11.**  **How likely are you to talk to him when you have a health problem or make an important health decision?** |
| --- | --- | --- | --- | --- | --- | --- | --- | --- | --- | --- | --- | --- | --- |
|  |  | **Full name (If inevitable, write down**  **nickname)** | **See relation legend** | Age | ① Men  ②  Women  N/A | ① Lower than elementary school  ② Elementary school  ③ Middle school  ④ High school  ⑤ Undergraduate school  ⑥ Graduate school or higher  N/A | ① Yes  ② No  N/A | ① Seoul ② Busan  ③ Daegu ④ Incheon  ⑤ Gwangju ⑥ Daejeon  ⑦ Ulsan ⑧ Gyeonggi  ⑨ Gangwon ⑩ Chungbuk  ⑪ Chungnam ⑫ Jeonbuk  ⑬ Jeonnam ⑭ Gyeongbuk  ⑮ Gyeongnam Jeju  ◯17 Sejong  Aborad N/A | ( ) years | ① Everyday  ② Several times a week  ③ Once a week  ④ Once every 2 weeks  ⑤ Once a month  ⑥ Several times a year  ⑦ Once a year  ⑧ Less than once a year  N/A | ① Everyday  ② Several times a week  ③ Once a week  ④ Once every 2 weeks  ⑤ Once a month  ⑥ Several times a year  ⑦ Once a year  ⑧ Less than once a year  N/A | ① Not very close.  ② Somewhat close.  ③ Close.  ④ Very close.  N/A | ① Very likely.  ② Somewhat likely.  ③ Not likely.  N/A |
| **Spouse** | 1 |  | 1 |  |  |  |  |  |  |  |  |  |  |
| **People you frequently talk to,**  **up to 5 people.** | 2 |  |  |  |  |  |  |  |  |  |  |  |  |
|  | 3 |  |  |  |  |  |  |  |  |  |  |  |  |
|  | 4 |  |  |  |  |  |  |  |  |  |  |  |  |
|  | 5 |  |  |  |  |  |  |  |  |  |  |  |  |
|  | 6 |  |  |  |  |  |  |  |  |  |  |  |  |
| **Closest individual to you, other than**  **1-6.** | 7 |  |  |  |  |  |  |  |  |  |  |  |  |

| **Supplementary Table 2. OR for K-MMSE score of over 24 in Cardiovascular and Metabolic Diseases Etiology Research Cohort (CMERC) participants** | | | | | | | | |
| --- | --- | --- | --- | --- | --- | --- | --- | --- |
| **Men** | **(N=2678)** | | | | | | |  |
|  | **OR** |  | **95% CI** | | |  | **p-value** |  |
| **size (0-6)** | 1.071 | (0.976-1.176) | | | | | 0.1485 |  |
| **intimacy (1-4)** | 1.228 | (1.039-1.451) | | | | | 0.0161 |  |
| **frequency (1-8)** | 1.213 | (1.062-1.384) | | | | | 0.0043* |  |
|  |  |  |  |  |  |  |  |  |
| **Women** | **(N=4252)** | | | | | | |  |
|  | **OR** |  | **95% CI** | | |  | **p-value** |  |
| **size (0-6)** | 1.088 | (1.027-1.154) | | | | | 0.0044* |  |
| **intimacy (1-4)** | 1.066 | (0.944-1.204) | | | | | 0.3035 |  |
| **frequency (1-8)** | 1.076 | (0.998-1.159) | | | | | 0.0570 |  |
| The model is adjusted for age, household income, education level, marital status, living arrangement, occupation, physical activity(MET-minutes), BDI score, cigarette use, alcohol consumption and study settings. | | | | | | | | |
|  |  |  |  |  |  |  |  |  |
|  |  |  |  |  |  |  |  |  |
|  |  |  |  |  |  |  |  |  |
| K-MMSE, Korean version of Mini-Mental State Examination; OR, odds ratio, 95% CI, 95% confidence interval  *Significant in Bonferroni correction | | | | | | | | |
